# Increased Anxiety-Related Behavior, Impaired Cognitive Function and Cellular Alterations in the Brain of Cend1-deficient Mice

**DOI:** 10.3389/fncel.2018.00497

**Published:** 2019-01-29

**Authors:** Katerina Segklia, Antonios Stamatakis, Fotini Stylianopoulou, Alexandros A. Lavdas, Rebecca Matsas

**Affiliations:** ^1^Laboratory of Cellular and Molecular Neurobiology—Stem Cells, Department of Neurobiology, Hellenic Pasteur Institute, Athens, Greece; ^2^Biology-Biochemistry Lab, Faculty of Nursing, School of Health Sciences, National and Kapodistrian University of Athens, Athens, Greece

**Keywords:** GABAergic interneurons, neurogenesis, ganglionic eminence, somatosensory cortex, basolateral amygdala, dentate gyrus

## Abstract

Cend1 is a neuronal-lineage specific modulator involved in coordination of cell cycle exit and differentiation of neuronal precursors. We have previously shown that Cend1^−/−^ mice show altered cerebellar layering caused by increased proliferation of granule cell precursors, delayed radial granule cell migration and compromised Purkinje cell differentiation, leading to ataxic gait and deficits in motor coordination. To further characterize the effects of Cend1 genetic ablation we determined herein a range of behaviors, including anxiety and exploratory behavior in the elevated plus maze (EPM), associative learning in fear conditioning, and spatial learning and memory in the Morris water maze (MWM). We observed significant deficits in all tests, suggesting structural and/or functional alterations in brain regions such as the cortex, amygdala and the hippocampus. In agreement with these findings, immunohistochemistry revealed reduced numbers of γ amino butyric acid (GABA) GABAergic interneurons, but not of glutamatergic projection neurons, in the adult cerebral cortex. Reduced GABAergic interneurons were also observed in the amygdala, most notably in the basolateral nucleus. The paucity in GABAergic interneurons in adult Cend1^−/−^ mice correlated with increased proliferation and apoptosis as well as reduced migration of neuronal progenitors from the embryonic medial ganglionic eminence (MGE), the origin of these cells. Further we noted reduced GABAergic neurons and aberrant neurogenesis in the adult dentate gyrus (DG) of the hippocampus, which has been previously shown to confer spatial learning and memory deficits. Our data highlight the necessity of Cend1 expression in the formation of a structurally and functionally normal brain phenotype.

## Introduction

During brain development neural stem and progenitor cells located in proliferative zones produce a variety of neurons, thereby generating the diversity and complexity of neuronal phenotypes present in the adult brain (Jessell, 2000; Marquardt and Pfaff, 2001; Bertrand et al., 2002; Cheng et al., 2004; Götz and Huttner, 2005; Arlotta et al., 2008; Achim et al., 2014; Götz et al., 2016). Neural progenitors proliferate in the ventricular and subventricular zones of the developing forebrain, then exit the cell cycle and differentiate as they migrate away from these zones. During this process coordinated regulation of cell cycle exit and differentiation is essential for generation of the appropriate number of neurons and formation of the correct structural and functional connections of neuronal circuits. Previous studies have shown that the differentiation of neuronal progenitors is intimately connected with the regulation of the cell cycle and that the two processes are under coordinate control (Qian et al., 2000; Ohnuma and Harris, 2003; Hardwick et al., 2015).

Cend1 is a neuronal-lineage specific modulator involved in timely coordination of cell cycle exit and differentiation of neuronal precursors (Koutmani et al., 2004; Georgopoulou et al., 2006; Politis et al., 2007a,b). Its expression is low, but clearly detectable, in precursor cells and is up-regulated upon neuronal differentiation, suggesting a functional role in the precursor to neuron switch. In agreement, gain- and loss-of-function experiments in neural stem/ progenitor cells showed that Cend1 prompts neuronal precursors to exit the cell cycle and differentiate to a neuronal phenotype (Katsimpardi et al., 2008; Makri et al., 2010). Further, genetic ablation of Cend1 in mice resulted in irregularities in cerebellar lamination caused by increased proliferation of granule cell precursors, delayed radial migration of granule cells and compromised Purkinje cell differentiation, particularly evident in stunted arborization of their dendritic tree (Sergaki et al., 2010). These alterations were associated with ataxic gait and deficits in motor coordination in Cend1^−/−^ mice.

To examine further the consequences of Cend1 genetic ablation, we determined herein a range of behaviors, including anxiety and exploratory behavior in the elevated plus maze (EPM), associative learning in fear conditioning, and spatial learning and memory in the Morris water maze (MWM). We observed significant deficits in all tests, suggesting structural and/or functional alterations in brain regions such as the cortex, amygdala and the hippocampus. In concurrence, immunohistochemistry revealed reduced numbers of γ-aminobutyric acid (GABA) GABAergic interneurons in the adult cerebral cortex and the amygdala, most notably in the basolateral nucleus, as well as aberrant neurogenesis in the dentate gyrus (DG) of the hippocampus.

GABAergic interneurons comprising about one fifth of the total neuronal population in the adult cortex, play a pivotal role in cortical circuitry and activity. They provide the principal source of inhibition to cortical circuits and regulate the activity of excitatory projection neurons (Lehmann et al., 2012; Kelsom and Lu, 2013). Previous studies have indicated that changes in the number, distribution and function of cortical interneurons are associated with a variety of severe neurological disorders, including autism, schizophrenia, epilepsy and anxiety (Matsuda et al., 2011; Le Magueresse and Monyer, 2013; Catavero et al., 2018). Cortical interneurons originate from the ganglionic eminence (GE), mostly the medial part medial GE (MGE), which is a distinct domain of the subpallial ventricular area, and migrate tangentially to reach the developing cortex where they reside (Marín and Rubenstein, 2003; Nakajima, 2007). The cellular and molecular mechanisms involved in interneuron generation and migration from their site of origin to the cortex have been studied extensively. The list of molecules involved includes both cell intrinsic cues—permissive or instructive—and cell extrinsic factors- attractive or repulsive (Crair, 1999; Vanderhaeghen and Polleux, 2004). Interestingly, cell cycle progression/exit of GE precursors has been linked to their normal migratory behavior (Vidaki et al., 2012) rendering Cend1 a potential player in this process.

Apart from cortical interneurons, the GE gives rise to GABAergic interneuron subtypes that contribute to the mammalian amygdala (Waclaw et al., 2010). This region is subdivided to the lateral, basolateral, and central amygdala and has been identified as a fundamental anatomic area of the circuitry processing fear conditioning (LeDoux, 2000, 2003). An important function of this region is to control behaviors that are related to fear and anxiety, in concert with the hippocampus and the cerebral cortex (Roozendaal et al., 2009), whose GABAergic interneurons also originate at the GEs (Nery et al., 2002). Notably, it has been reported that within this circuit inhibitory interneurons play a vital role in fear-related behaviors (Ehrlich et al., 2009).

In the present study we identified functional and cellular aberrations in Cend1^−/−^ mice, likely associated with reduced numbers of GABAergic, but not of glutamatergic, cells. We established that the paucity of GABAergic interneurons in the adult Cend1^−/−^ cortex and amygdala correlated with increased proliferation and apoptosis as well as with reduced migration of neuronal progenitors from the embryonic GE from which these cells originate. We further noted reduced hippocampal GABAergic neurons and aberrant neurogenesis in the adult DG of the hippocampus, both implicated in learning and memory processes. In the DG new neurons are produced continuously in the adult brain and become functionally integrated into pre-existing neuronal circuits thus contributing to behaviorally relevant neuronal assemblies (Eriksson et al., 1998; Kempermann et al., 2004; Parent, 2007; Trouche et al., 2009; Song et al., 2012; Nicola et al., 2015). An increasing number of studies indicates that adult hippocampal neurogenesis is functionally associated with hippocampal-dependent learning (Shors et al., 2001; Leuner et al., 2004; Snyder et al., 2005; Winocur et al., 2006; Kee et al., 2007; Kempermann et al., 2015). Moreover, adult-generated hippocampal neurons are involved in spatial memory processes such as acquisition or retrieval, especially while still in an immature stage when they possess increased plasticity (Ramirez-Amaya et al., 2006; Tashiro et al., 2007; Abrous and Wojtowicz, 2015). Our analysis showed an activation of the earlier stages of adult hippocampal neurogenesis in Cend1^−/−^ mice, accompanied by increased cell apoptosis. These events may be causally related to a parallel decrease in local parvalbumin (PV) interneurons, which are known to suppress neural stem cell activation (Song et al., 2012), but also to support the survival of newborn neurons (Song et al., 2013).Taken together, our data highlight the requirement for Cend1 expression in the formation of a structurally and functionally normal phenotype.

## Materials and Methods

### Animals

All international guidelines for animal care and use were applied in strict compliance with the European and National Laws for Laboratory Animal Use (Directive 2010/63/EU and Greek Law 56/2013), according to FELASA recommendations for euthanasia and the Guide for Care and Use of Laboratory Animals of the National Institutes of Health. All protocols were approved by the Animal Care and Use Committee of the Hellenic Pasteur Institute (Animal House Establishment Code: EL 25 BIO 013, License No. 4550/ 11-07-2014). The Cend1^−/−^ colony was maintained in the heterozygous state by backcrossing to C57BL/6J mice (Sergaki et al., 2010). Homozygous Cend1−/− adult animals or E14.5- E16.5 embryos were produced by intercrossing heterozygous Cend1+/– mice. Genotyping was performed by PCR using two different sets of oligonucleotide primers: Forward 5′-CTAGAGAATTCAGGGAATTGGGGATG-3′ and reverse 5′-AGTGTTGGACTCGTCCTCCTCTG-3′ primers were used to amplify a 500-bp band within the Cend1 coding region present in wild-type (wt) and heterozygous animals and absent in knock-out mice. Forward 5′-CCGACGGCACGCTGATTGA-3′ and reverse 5′-GCTCCGCCGCCTTCATACTG-3′ primers derived from the lacZ gene in the pZ-lacz vector were used to amplify a 500-bp band present in heterozygous and knock-out mice and absent in wt animals.

### Behavioral Analyses

Three different cohorts of animals were used for the behavioral analyses of Cend1^−/−^ adult male mice: one for the EPM (10^+/+^ and 15^−/−^ animals), one for the cued fear conditioning (8^+/+^ and 8^−/−^ animals) and one for the MWM test (11^+/+^ and 14^−/−^ animals).

### Analysis of Anxiety Related Behavior

#### Elevated Plus Maze (EPM)

Mice were assessed between 09:00–12:00 h, during the light period of the daily cycle, in a room with moderate illumination of 150 lux. The apparatus encompassing two open and two closed arms, was elevated 60 cm above the ground. It was made of plastic, with a black-colored floor and gray–colored walls, 40 cm in height for the closed compartments. The central platform was 10 × 10 cm in size and the arms 50 × 10 cm in size. Each animal was placed in the center of the apparatus and it faced an open arm. Each animal was left to explore the EPM for 5 min and its movements were digitally recorded. For an animal to be considered within (entry) or outside (exit) an arm, all its limbs should be in the same arm. The following parameters were quantified independently by two investigators “blind” as to the group that animals belonged to: number of entries and time spent in both open arms, number of entries and time spent in both closed arms.

### Behavioral Analysis of Learning and Memory Function

#### Cued Fear Conditioning

Before training, each animal was habituated for 3 min to the conditioning apparatus (black-colored box, Pansystems, 25 × 25 × 27 cm). After the habituation period, the animal was exposed to a tone (8 kHz, 76 dB) for 30 sco-terminating with an electric foot shock (intensity: 0.5 mA; duration: 1 s). This pairing was repeated three times. Assessment of cued fear memory was performed 8 days after training, in a modified conditioning apparatus: the floor grid metal bars delivering the shock were covered with a white cardboard and cut-out circles and rectangles were mounted on the walls of the conditioning box. In addition, novel olfactory cues (rose odor) had been introduced to the testing box. Each mouse received three 30-s tone presentations without shock, separated by 60 s intervals. Their behavior was analyzed off-line independently by two investigators “blind” as to the group the animals belonged to. Freezing was defined as absence of any other movement except the respiratory ones. The average time of freezing during the 30 s tones, was calculated for each animal.

#### Morris Water Maze Test (MWM)

The allocentric version of the MWM was employed, using a circular pool of 140 cm diameter. The pool was filled with water (24 ± 1°C); in order to make the water opaque, a non-toxic, water-soluble white dye was added to the water. Mice were trained for 5 consecutive days and 24 h later they were exposed to a memory probe trial. During training, mice had to locate a hidden platform (8 cm × 10 cm made of transparent plastic) positioned 1 cm under the water surface; the position of the platform was fixed relative to extra-maze visual cues. Each day animals were exposed to four trials (each trial had a maximum duration of 60 s), separated by 15 min intervals. In each trial the starting position was different, in a pseudorandom order. Each trial ended when the animal found the platform or after 60 s in which case the animal was placed by the experimenter on the platform. In either case, it remained on the platform for 20 s and then it was returned in its home cage. Memory probe trial took place 24 h after the last training trial. For the probe trial, the platform was removed, the animal entered the water maze from a point opposite to the quadrant of the maze were the platform used to be during training and it was allowed to explore the maze for 60 s. The Noldus Ethovision system (Ethovision 3.0, Noldus Information Technologies, Wageningen, Netherlands) was use for the documentation and analyses of the behavior of mice during the training and memory probe trials. For training, for each mouse latency (time in sec to mount the platform) was determined for each trial and an average was calculated for each day. For the probe trial, we calculated the time spent in the target quadrant as well as that spent in the opposite quadrant. In order to exclude the possibility that any learning or memory deficits observed in the MWM were due to deficits in motor activity, swim speed (cm/sec) was also calculated.

### Tissue Preparation and Immunofluorescence Procedures

Mice (3–4 per genotype) were euthanized by isoflurane inhalation and perfused transcardially with 4% paraformaldehyde fixative in phosphate-buffered saline (PBS). Following dissection, brains were post-fixed at 4°C overnight and cryoprotected for 2 days at 4°C in sucrose (30% w/v in PBS). Tissues were then embedded in O.C.T. compound (VWR Chemicals) and frozen or in some cases processed for vibratome sectioning (50 μm), as indicated. Series of coronal cryosections 20 μm-thick were cut and kept at −20°C until processed for immunohistochemistry. After thawing sections were treated for 1 h with 5% normal donkey serum (NDS) to block non-specific binding sites and permeabilized with 0.2% v/v Triton X-100 in PBS. Overnight incubation with primary antibodies diluted in 2.5% NDS in PBS at 4°C was followed by incubation with secondary antibodies for 2 h at room temperature. Primary antibodies used were: rabbit polyclonal anti-GABA (1:100; Sigma) for GABAergic neurons; rat monoclonal anti-somatostatin (anti-Sst; 1:1,000; Millipore) for GABAergic interneuron subtype; mouse monoclonal anti-Satb2 (1:300; Abcam) for glutamatergic interneurons; rabbit polyclonal anti-calbindin (1:1,000; Chemicon) and rabbit polyclonal anti-PV (1:1,000; Swant) for calcium binding proteins; anti-phosphohistone 3 (PH3; 1:500, Upstate) and mouse monoclonal anti-Ki67 (1:400; Beckton Dickinson) for mitotic and proliferating cells, respectively; rabbit polyclonal anti-caspase 3 (1:400; Cell Signaling) for apoptosis; mouse monoclonal anti-glial fibrillary acidic protein (GFAP, 1:500; Sigma) for parenchymal astrocytes and neural stem cells in the subgranular layer (SGL) of the DG; rabbit polyclonal anti-Sox2 (1:200; Abcam) for neural stem and progenitor cells in the subgranular zone (SGZ) of the DG; goat polyclonal anti-doublecortin (DCX; 1:50; Santa Cruz); mouse monoclonal anti-NeuN (1:300; Chemicon) and rabbit polyclonal anti-Prox1 (1:200; Abcam) for newborn and mature DG neurons; rabbit polyclonal anti-Iba1 (1:200; Wako) and rat monoclonal anti-CD68 (1:150; Serotec) for total and activated microglia/macrophages; mouse monoclonal anti-Mash1 (1:50; BD Pharmigen) for identification of GABAergic interneuron precursors in the GEs. Secondary antibodies conjugated to Alexa Fluor 488 or 546 and TO-PRO-3 (1:1,000) used to visualize cell nuclei were from Molecular Probes. Sections were mounted on Prolong Gold antifade (Molecular Probes) and confocal microscopy was performed in Leica TCS SP and Leica TCS-SP5II confocal microscopes.

### Image Analysis

#### Cell Counts on Embryo and Adult Brain

For each animal, digital images from 3–4 brain sections were acquired in a confocal microscope and the number of cells positive for a specific marker were counted using ImageJ areas of interest, “blindly” as to the group animals belonged to, and setting the threshold at a constant value. The brain areas analyzed include the adult somatosensory cortex, the basolateral amygdala (BLA) and the hippocampal DG as well as the embryonic GEs. Counts were averaged from three fields per area and per animal (*n* = 3 mice per genotype).

#### Fluorescence Intensity

For evaluation of GFAP expression in the parenchyma of the DG, fluorescence intensity (pixels) was measured as previously described (Papastefanaki et al., 2015; Terzenidou et al., 2017). Briefly, single channel stacks of confocal images were acquired under the same settings (constant gain and offset values, 3× averaging, 1024 × 1024 resolution, 1-μm step size). Quantification of fluorescence intensity was performed using ImageJ software by a blind observer, after free-hand selection of the region of interest (ROI) and setting the threshold at a constant value. Measurements on each single image of the confocal stack were added up and normalized to the area of the ROI. Three animals were analyzed per genotype and the values from three sections were averaged per mouse.

#### Automated Calculation of the Density of Satb2^+^ Cells

In the first step of analysis, the Imaris “spots” module was used to calculate automatically the total number of nuclei (estimated diameter >6 μm) in the area of the somatosensory cortex. In the second step, only the nuclei that were positive for Satb2 were selected, using the “filter” module of spots (filter type: max intensity of green channel for Satb2). Finally, the number of Satb2^+^ cells was calculated as a percentage of total nuclei, and setting the threshold at a constant value. Counts were averaged from three fields per area and per animal (*n* = 3 mice per genotype).

### Preparation of Organotypic Slice Cultures

Slice cultures were prepared as previously described (Lavdas et al., 1999; Kouroupi et al., 2010). Briefly, pregnant C57BL6 mice at two different stages of gestation (E14, *n* = 3; E16, *n* = 3) were euthanized by isoflurane inhalation. The fetuses were immediately removed and immersed in sterile HBSS at 4°C containing 6.5 mg/ml glucose. All following procedures were performed under sterile conditions. Brains were removed and embedded in 3% agarose/ 0.1 M PBS, pH 7.2. Coronal slices (400 μm-thick) were cut using a Leica vibrating microtome and kept for 45 min at 4°C in HBSS/glucose to allow for decline of enzymatic activity released by damaged cells. Slices were placed onto millicell CM membranes in 30 mm Petri dishes containing 1 ml of DMEM/F12 supplemented with 6.5 mg/ml glucose/0.1 mM glutamine/50 mg/ml penicillin/streptomycin/10% FCS. After 1 h the medium was changed to Neurobasal supplemented with B27 (1:50) and N2 (1:100) containing 6.5 mg/ml glucose/0.1 mM glutamine/50 mg/ml penicillin/streptomycin.

### Administration of Fluorescent Tracer

To investigate neuronal migration, we used a glass micropipette to place DiI crystals (Molecular Probes; Lavdas et al., 1999; E14, *n* = 3; E16, *n* = 5) on one hemisphere of cultured slices. After tracer placement, slices were cultured in Neurobasal medium for a further 48 h and were then fixed for 3 h in 4% formaldehyde in PBS. Slices were subsequently rinsed in PBS, mounted on microscope slides, and observed with a laser-scanning confocal microscope (Leica TCS SP).

### Statistical Analyses

The effect of genotype on the latency to reach the platform during the learning period in the MWM was assessed using one-way ANOVA with repeated measures (day) and genotype as the independent factor. The effect of genotype on time spent in the target and opposite quadrants of the MWM during the long-term memory probe trial was assessed using a two-way ANOVA with genotype and quadrant as independent factors. In the case of significant interactions, univariate *F* tests were used to evaluate the main effects. Finally, the effect of genotype on all other behavioral parameters was assessed using one-way ANOVA, with genotype as the independent factor. For statistical comparisons of the immunohistochemical data between genotypes the two-tailed unpaired Student’s *t-test* was used. All statistical analyses were performed using the statistical software SPSS 21 for Windows. Values are expressed as mean ± standard error of mean (SEM) and significance is defined as *p < 0.05; **p < 0.01; ***p < 0.001 for genotype differences and ### *p* < 0.001 for the difference in the time spent in the target and opposite quadrants during the probe trial of MWM.

## Results

### Cend1^−/−^ Mice Exhibit Severe Behavioral Deficits

**Figure 1:**
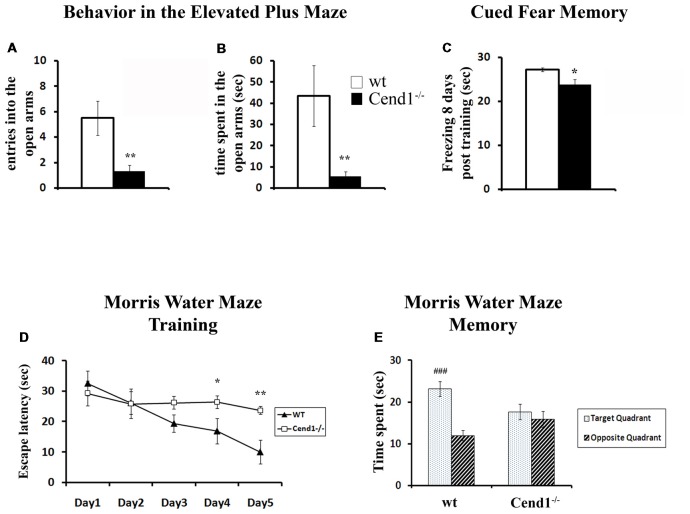
Behavioral deficits in adult Cend1^−/−^ mice. Cend1^−/−^ mice show increased anxiety as assessed in the elevated plus maze (EPM) task, making fewer entries **(A)** and spending less time in the open arms of the maze **(B)** than their wild-type (wt) littermates (*n* = 10 for wt mice and *n* = 15 for Cend1^−/−^ mice). Cend1^−/−^ mice show a mild deficit in cued fear memory 8 days following fear conditioning **(C)** as compared to wt animals (*n* = 8 for both genotypes), as well as severe cognitive deficits in the Morris water maze (MWM) test, both during learning **(D)** and in the memory trials (**E**; *n* = 11 for wt mice and *n* = 14 for Cend1^−/−^ mice). Values represent mean ± standard error of mean (SEM); *P < 0.05; **P < 0.01; ###, *P* < 0.001.

To investigate the effects of Cend1 genetic ablation, we exposed Cend1^−/−^ mice to a repertoire of behavioral tests. First, we assessed anxiety related behavior in the EPM, where Cend1^−/−^ mice spent less time in and made fewer entries into the open arms compared to wt animals, indicative of higher levels of anxiety. More specifically, for both the time spent in the open arms and the number of entries into the open arms, statistical analysis revealed a significant genotype effect: *F*_(1,24)_ = 9.994, *p* = 0.004 and *F*_(1,24)_ = 12.621, *p* = 0.002, respectively (Figures 1A,B). Notably, no difference was observed in the number of entries into the closed arms (*F*_(1,24)_ = 0.517, *p* = 0.479).

Cend1^−/−^ mice also showed a mild but statistically significant deficit in cued fear memory 8 days following fear conditioning (*F*_(1,15)_ = 8.684, *p* = 0.011; Figure 1C). It should be noted that during fear conditioning training, both Cend1^−/−^ and wt mice oriented towards the source of the tone (i.e., could detect the tone) and they showed the normally expected response to the noxious stimulus of the electric foot shock.

Finally, mice were assessed for spatial learning and memory in the MWM, a hippocampal-dependent task which is also used to evaluate possible damage to cortical regions of the brain (D’Hooge and De Deyn, 2001). Adult mice went through a training phase during which they were evaluated for their learning ability by measuring the latency to find a hidden platform. All mice, either wt or Cend1^−/−^, had a normal swimming speed and no difficulty to climb on the submerged escape platform. It should be mentioned that similarly to our observations, in other models of ataxic mice, ataxia did not affect the swimming speed of the animals possibly due to the nature of this measurement: gait-impaired animals can apparently compensate in the water with other body movements or with extra hind-limb kicks (Lalonde and Botez, 1986; Savvaki et al., 2008; Jayabal et al., 2015).

Nevertheless, a significant day × genotype interaction was observed on the latency to find the hidden platform (*F*_(4,92)_ = 3.955, *p* = 0.005). Further analysis indicated that wt animals showed the normal improvement in their performance across days (progressively reduced latency to find the hidden platform) while Cend1^−/−^ mice failed to do so. More specifically, on days 4 and 5 of training, Cend1^−/−^ animals showed statistically significant higher latencies to reach the hidden platform compared to wt mice (*p* = 0.045 for Day 4 and *p* = 0.005 for Day 5; Figure 1D). In the long-term memory probe trial (24 h after the last training trial), statistical analysis revealed a significant quadrant × genotype interaction on the time spent in the target and opposite quadrants (*F*_(1,49)_ = 8.382, *p* = 0.006; Figure 1E). As expected, given their poor learning performance, Cend1^−/−^ mice showed no preference for the target quadrant (*post hoc* test *p* = 0.452), while wt animals exhibited a clear preference spending more time in the target quadrant as compared to the opposite one (*post hoc* test *p* < 0.001).

### Cend1^−/−^ Mice Have Reduced Number of Interneurons in the Adult Cortex and Amygdala

**Figure 2:**
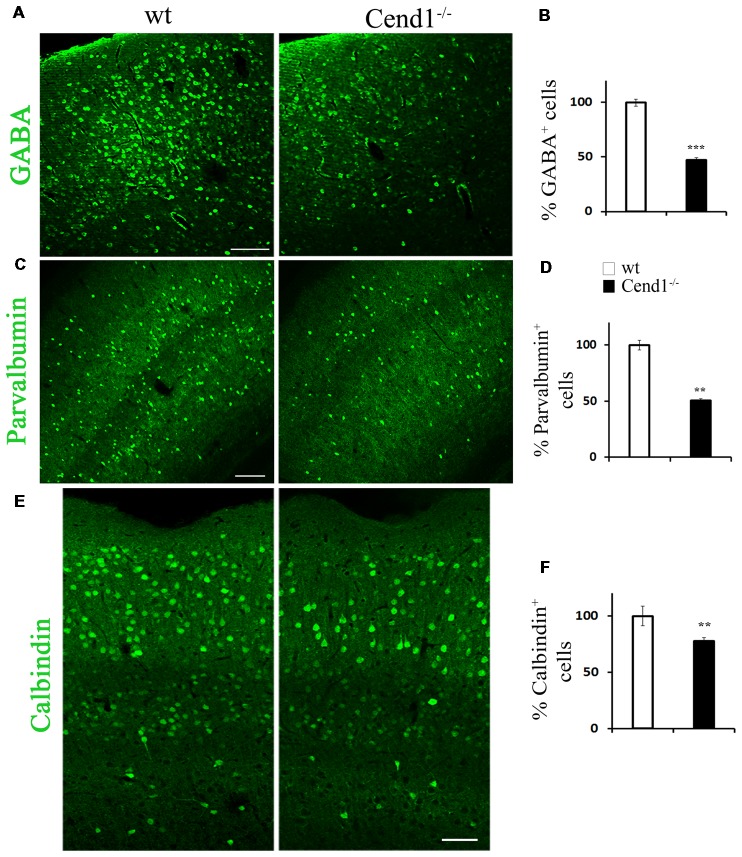
Reduced interneurons in the adult cortex of Cend1^−/−^ mice. Cryostat sections of adult somatosensory cortex immunostained for γ-aminobutyric acid (GABA; **A**), parvalbumin (PV; **C**) and calbindin **(E)**. Percentage of GABA^+^ interneurons is reduced by half in Cend1^−/−^ mice by comparison to wt animals **(B)**. This is mainly reflected in reduction of PV^+^ cells **(D)** and to a lesser extent in reduction of calbindin^+^ cells **(F)** in Cend1^−/−^ mice as compared to wt animals (*n* = 3 per genotype). Values represent mean ± SEM. Scale bars **(A,C)**, 100 μm; **(E)**, 60 μm; **P < 0.01; ***P < 0.005.

**Figure 3:**
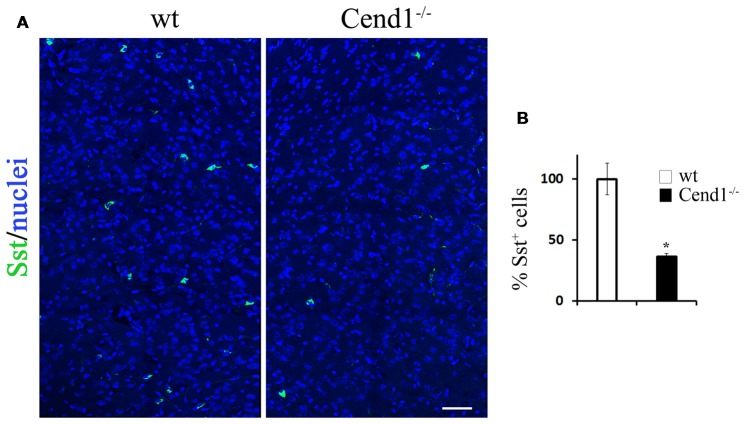
Decreased somatostatin (Sst)-expressing interneurons in the adult cortex of Cend1^−/−^ mice. Confocal images of cryostat sections of adult somatosensory cortex immunostained for Sst **(A)** and quantification of Sst^+^ cells in Cend1^−/−^ mice as compared to wt animals (**B**; *n* = 3 per genotype). Values represent mean ± SEM. Scale bar **(A)**, 50 μm; *P < 0.05.

We next sought to investigate whether the behavioral phenotype of Cend1^−/−^ mice is accompanied by cellular changes in specific brain regions that are functionally relevant. It has been shown that increased levels of anxiety are related to a reduction in the GABAergic population, resulting in decreased inhibitory neurotransmission in the cortex (Löw et al., 2000). In agreement, immunofluorescence labeling for the neurotransmitter GABA in coronal cortical sections from adult mice revealed a decrease in immunolabeled cells in Cend1^−/−^ mice as compared to wt, particularly in the somatosensory cortex (Figure 2A). Quantification demonstrated a specific, statistically significant reduction in the density of GABAergic neurons (53% reduction, *P* = 0.0007) in this region (Figure 2B) whilst the density of glutamatergic neurons identified by Satb2 immunofluorescence, was not changed in Cend1^−/−^ mice (Supplementary Figure S1). We further examined the presence of interneuron subtypes in the adult cortex by immunofluorescence for the calcium-binding proteins PV and calbindin. We noted a prominent reduction (49%, *P* = 0.003) in the number of PV-expressing interneurons (Figures 2C,D) of Cend1^−/−^ mice as compared to their wt littermates, as well as a significant reduction (22% reduction, *P* = 0.008) in the number of calbindin^+^ interneurons (Figures 2E,F). Finally, Sst-expressing interneurons that have a much lower abundance than the other two interneuron subtypes, were also significantly reduced (63% reduction, *P* = 0.031; Figure 3).

**Figure 4:**
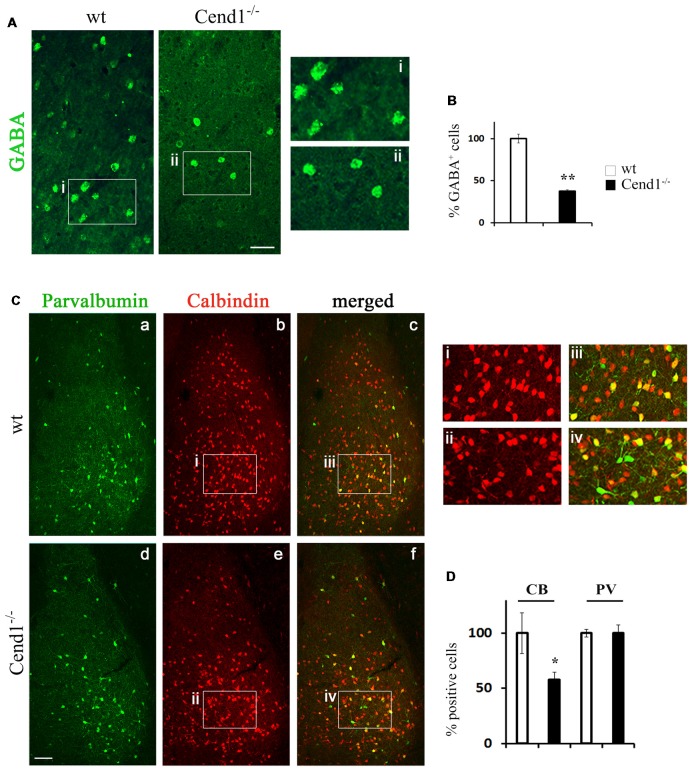
Reduced interneurons in the adult amygdala of Cend1^−/−^ mice. Confocal images of vibratome sections of adult amygdala immunostained for GABA **(A)** and quantification of GABA^+^ cells in Cend1^−/−^ mice as compared to wt animals **(B)**. The insets in **(A)** are shown at higher magnification in (Ai-Aii). **(C)** Double immunofluorescence labeling for PV (green; **Ca,d**) and calbindin (red; **Cb,e**) followed by confocal imaging shows partial overlap of the two populations **(Cc,f)**. The insets in **(C)** are shown at higher magnification in (Ci-Civ). **(D)** Quantification shows that PV^+^ cells do not differ between wt and Cend1^−/−^ mice while calbindin^+^ cells are reduced almost by half in Cend1^−/−^ mice (*n* = 3 mice per genotype). Values represent mean ± SEM. Scale bar 40 μm **(A)**, 100 μm **(Cd)**; *P < 0.05; **P < 0.01.

Apart from the cortex, the behavioral phenotype of Cend1^−/−^ mice pointed to possible cellular alterations in the amygdala, a region associated with fear and anxiety-related behavior along with the cerebral cortex. Previous studies have shown that a reduction in the GABAergic population residing in the amygdala (Waclaw et al., 2010) correlated with increased levels of anxiety (LeDoux, 2003), with the BLA and central nuclei being especially involved in the amygdalar circuitry that mediates control of anxiety (Tye et al., 2011). Based on these observations, we examined by immunofluorescence labeling the GABAergic population in the amygdala of Cend1^−/−^ and wt mice and found a severely compromised population of GABAergic neurons (62% reduction, *P* = 0.008) in the mutant mice (Figures 4A,B). Examination of interneuron subtypes revealed a statistically significant decrease in the number of calbindin^+^ cells in Cend1^−/−^ mice (41% reduction, *P* = 0.024) as compared to wt (Figures 4Ca–f,D), particularly in the basolateral nucleus (BLA). However, unlike the somatosensory cortex, we did not observe a similar reduction in the PV^+^ population in this brain area (Figures 4Ca,d,D).

### Reduced Migration of MGE-derived GABAergic Progenitors Is Associated With Altered Cell Proliferation and Apoptosis in Cend1^−/−^ Mouse Embryos

**Figure 5:**
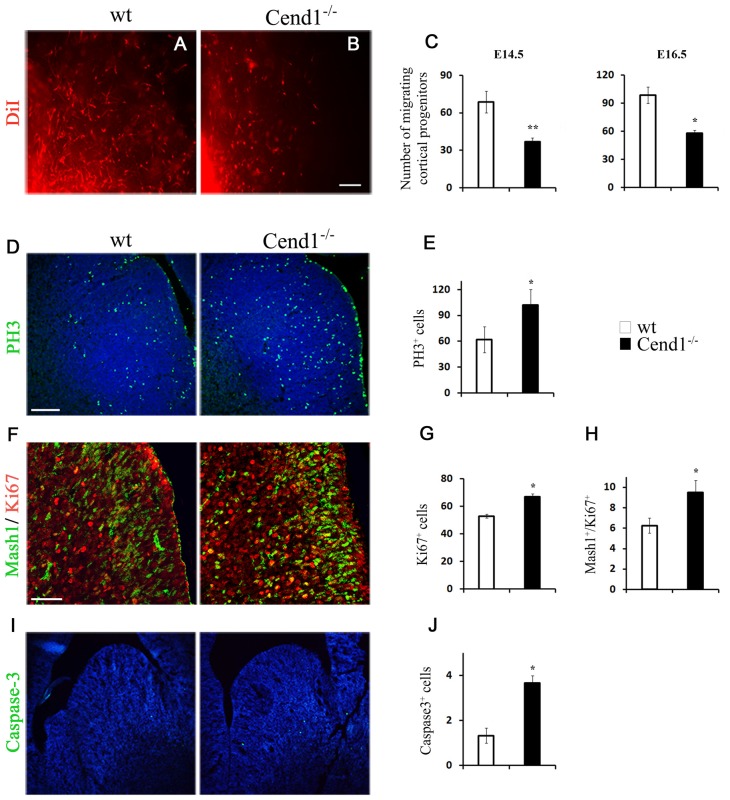
Reduced migration and altered cell proliferation and apoptosis in Cend1^−/−^ mouse embryos. **(A,B)** Live images of DiI-labeled wt **(A)** and Cend1^−/−^ **(B)** organotypic slices from E16.5 mouse forebrain after 2 days *in vitro*. At the bottom left, shown is the edge of the DiI crystal placed on the medial ganglionic eminence (MGE) to label migrating GABAergic interneuron precursors. **(C)** Quantification of DiI-labeled cells at the beginning of the “stream” adjacent to the MGE in E14.5 and E16.5 slices reveals significantly decreased numbers of migrating cortical progenitors in Cend1^−/−^ mice [E14.5: 68.7 ± 8.3 cells in wt (*n* = 4) vs. 37 ± 2 in Cend1^−/−^ mice (*n* = 3); E16.5: 98.4 ± 9.7 cells in wt (*n* = 5) vs. 58.0 ± 10.1 in Cend1^−/−^ mice (*n* = 4)]. **(D)** Immunofluorescence for the mitotic marker phosphohistone 3 (PH3) in cryostat sections of E14.5 MGE and quantification of labeled cells in **(E)**, show significantly increased proliferation in Cend1^−/−^ mice (62.0 ± 14.8 cells in wtvs.102.3 ± 24.7 in Cend1^−/−^ mice, *n* = 4 per genotype). **(F)** Double immunofluorescence labeling for Mash1 (green) and Ki67 (red) in cryostat sections of E14.5 MGE. Quantification of immunolabeled cells reveals a significant increase in the number of proliferating Ki67^+^ cells (**G**; 53.0 ± 1.5 in wt: vs. 67.0 ± 2.), as well as in cycling Mash1^+^/Ki67^+^ neuronal progenitors (**H**; 6.0 ± 0.7 in wt vs. 9.75 ± 0.85 in Cend1^−/−^ mice; *n* = 3 per genotype). Immunofluorescence **(I)** for the apoptotic marker caspase-3 and quantification in **(J)** shows a significant increase in cell death in Cend1^−/−^ mice (1.33 ± 0.33 in wt vs. 3.66 ± 0.33 in Cend1^−/−^ mice; *n* = 4 per genotype). Nuclei (blue) are visualized with TOPRO3 in **(D, I)**. Values represent mean ± SEM. Scale bars **(B, D)**, 100 μm; **(F)**, 50 μm; *P < 0.05; **P < 0.01.

To evaluate the origin of the cellular changes in the GABAergic population observed in the adult cortex and the amygdala of Cend1^−/−^ mice, we questioned what happens during development that could possibly lead to such a defect. To address this, we investigated the generation of GABAergic interneurons in the medial embryonic GE which gives rise to cortical GABAergic interneurons, but also to a population of interneurons of the adult amygdala. Fluorescent tracer experiments have previously shown that cells deriving from the MGE migrate to the developing cortex (Lavdas et al., 1999). To investigate whether Cend1 deletion affects the migratory behavior of MGE-derived interneurons, we used a similar approach by placing DiI crystals in the MGE of slice explants cultures prepared from the brains of mouse embryos at embryonic days E14.5 (Figure 5A) and E16.5 (Figure 5B). After further incubation for 48 h, DiI^+^ cells that had migrated outside the core of labeled cells were seen in both wt and Cend1^−/−^ mice. However, the numbers of migrating cortical progenitors were reduced almost by half in Cend1^−/−^ mice, at both developmental stages (for E14.5 *P* = 0.003 and for E16.5 *P* = 0.02; Figure 5C).

We then asked if the reduction in migrating progenitors is due to perturbed progenitor cell proliferation in the MGE. Immunofluorescence labeling of E14.5 coronal forebrain sections with the mitotic marker PH3 and quantification revealed a statistically significant increase (40% increase, *P* = 0.041) in the number of cells undergoing mitosis in the GE of Cend1^−/−^ mice when compared to their wt littermates (Figures 5D,E). Thus, in the absence of Cend1, fewer cells are likely to exit the cell cycle and start their trajectory to the cortex. We then examined the expression of Mash1 (Ascl1), a transcription factor with a pivotal role in the generation and specification of GABAergic interneurons (Castro et al., 2011; Xu et al., 2014). Double immunofluorescence labeling for Mash1 and the proliferation marker Ki67, revealed a significant increase in both the total number of proliferating Ki67^+^ cells (21% increase, *P* = 0.015; Figures 5F,G) as well as in the cycling Mash1^+^/Ki67^+^ neuronal progenitors (38% reduction, *P* = 0.014; Figures 5F,H) in the MGE of Cend1^−/−^ mice. Concomitantly, immunofluorescence labeling for activated caspase-3 as an apoptotic marker, revealed a statistically significant increase (41% increase, *P* = 0.035) in apoptotic cell death in the GE of Cend1^−/−^ mice as compared to wt littermates (Figures 5I,J). The combined phenomena of increased cellular proliferation in germinal zones accompanied by increased cell death and reduced migratory activity could contribute to the lower number of GABAergic interneurons in the cortex and amygdala of adult Cend1^−/−^ mice.

### Reduced Interneurons and Aberrant Neurogenesis in the Hippocampus of Adult Cend1^−/−^ Mice

**Figure 6:**
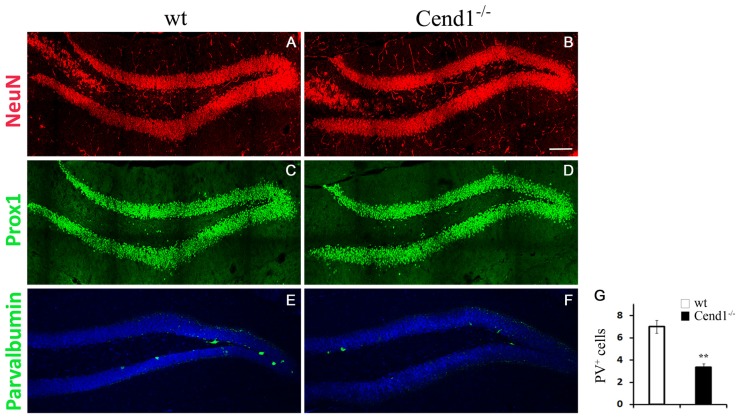
Decreased PV-expressing interneurons in the hippocampal dentate gyrus (DG) of adult Cend1^−/−^ mice. Cryostat sections immunostained for the mature neuronal marker NeuN **(A,B)** and the DG granule neuron marker Prox1 **(C,D)**, show similar expression patters between genotypes. Immunofluorescence labeling **(E,F)** and quantification **(G)** reveals a significant reduction in PV-expressing interneurons in the DG of Cend1^−/−^ mice (7.0 ± 0.5 PV cells in wt vs. 3.33 ± 0.33 in Cend1^−/−^ mice; *n* = 3 mice per genotype). Nuclei are visualized with TOPRO3. Values represent mean ± SEM. Scale bar **(B)**, 100 μm; **P < 0.01.

As adult hippocampal neurogenesis is intimately related to higher cognitive functions, most notably learning and memory processes (Kempermann et al., 2015), we examined if the cognitive deficit noted in Cend1^−/−^ mice is associated with cellular alterations in the SGZ of the adult DG, which constitutes the adult hippocampal neurogenic niche. GFAP^+^ and/or Sox2^+^ neural stem and progenitor cells residing in the SGZ of the DG generate proliferating DCX^+^ neuroblasts that give rise to post-mitotic DCX^+^ newborn neurons, which subsequently differentiate into granule neurons. Immunofluorescence for the general neuronal marker NeuN and the transcription factor Prox1 that specifically marks DG granule neurons, did not show any overt differences between genotypes (Figures 6A–D). However, there was a significant decrease in PV-expressing interneurons in the DG of Cend1^−/−^ mice (53% reduction, *P* = 0.0059; Figures 6E–G). Interestingly these local interneurons (basket cells), which are located in the immediate vicinity of the SGZ, have a dual function: on one hand they are known to suppress the activation of quiescent neural stem cells through GABA release, and on the other to control the survival of newborn neurons in the hippocampal neurogenic niche (Song et al., 2012, 2013; Saleem et al., 2013).

**Figure 7:**
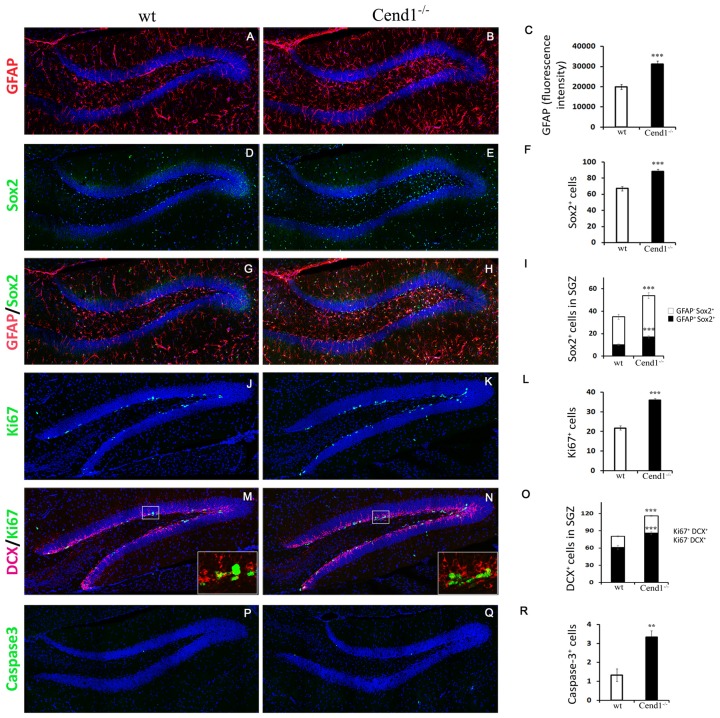
Aberrant neurogenesis in the hippocampus of adult Cend1^−/−^ mice. **(A–I)** Cryostat sections of the hippocampal DG double-labeled for GFAP (red) and Sox2 (green). Nuclei are seen with TOPRO3 (blue). Quantification of GFAP fluorescence intensity is shown in [**(C)**; 19904 ± 1242 arbitrary units in wt vs. 31284 ± 1502 in Cend1^−/−^ mice; *n* = 3 mice per genotype] and of the total number of Sox2^+^ cells in [**(F)**; 67.0 ± 2.7 vs. 88.3 ± 2.5 in Cend1^−/−^ mice; *n* = 3 mice per genotype]. The numbers of GFAP^+^/Sox2^+^ neural stem cells and GFAP^−^/Sox2^+^ precursor cells in the subgranular zone (SGZ) of the DG are quantified in **(I**; 10.1 ± 0.8 in wt vs. 17.2 ± 1.0 GFAP^+^/Sox2^+^ cells in Cend1^−/−^ mice and 24.83 ± 2.2 in wt vs. 36.66 ± 2.45 GFAP^−^/Sox2^+^ cells in Cend1^−/−^ mice; *n* = 3 per genotype). **(J,K)** Immunofluorescence labeling for the proliferation marker Ki67 and quantification **(L)** reveals a significant increase of Ki67^+^ cells in the SGZ of Cend1^−/−^ mice (21.8 ± 0.9Ki67^+^ cells in wt vs. 36.0 ± 0.9 in Cend1^−/−^ mice; *n* = 3 per genotype). Double immunofluorescence labeling for the neuroblast/newborn neuron marker doublecortin (DCX) and the proliferation marker Ki67 **(M,N)** and quantification **(O)** shows an increase in both proliferating neuroblasts and non-proliferating newborn neurons (19.4 ± 1.1 DCX^+^/Ki67^+^ cycling neuroblasts in wt vs. 30.0 ± 0.8 in Cend1^−/−^ mice; 60.9 ± 3.9 DCX^+^/Ki67^−^ newborn neurons in wt vs. 86.16 ± 2.54 in Cend1^−/−^ mice; *n* = 3 per genotype). **(P,Q)** Immunofluorescence labeling for the apoptotic marker activated caspase-3 and quantification **(R)**, shows a significant increase of cell death in the SGZ of Cend1^−/−^ mice (1.33 ± 0.33 caspase-3^+^ cells in wt vs. 3.33 ± 0.33 in Cend1^−/−^ mice; *n* = 3 per genotype). Values represent means ± SEM. Scale bar **(Q)**, 100 μm; **P < 0.01; ***P < 0.005.

To examine if the reduction in PV interneurons was paralleled by an increase in neural stem and precursor cells in the SGZ, we performed double labeling for GFAP and Sox2. Surprisingly, we noted an increase in GFAP immunoreactivity (37% increase, *P* = 0.0001; Figures 7A–C) as well as in the number of Sox2^+^ cells (24%, *P* = 0.0002) throughout the DG, particularly within the hilus, suggesting astroglial activation (Figures 7D–F). To check if this was a sign of astrogliosis accompanied by microgliosis and inflammation, we immunolabeled for the ionized calcium-binding adapter molecule 1 (Iba1) and CD68, to detect total and activated microglial cells, respectively. However, we could not find evidence for inflammatory astro- and/or microgliosis, as we did not detect any differences between genotypes (Supplementary Figure S2).

Local astrocytes play a key role in promoting neurogenesis (Kempermann et al., 2015). To address if the increased levels of astrocytes in concert with the reduction in local PV interneurons affected neurogenesis, we next quantified the numbers of GFAP^+^/Sox2^+^ neural stem cells and GFAP^−^/Sox2^+^ progenitor cells lying in the SGZ (Figures 7G–I). We found a significant increase of both cell types in Cend1^−/−^ mice (40%, *P* = 0.0004 and 35%, *P* = 0.0002, respectively), suggesting an expansion of the neural stem/progenitor cell pool. This was further supported by an increase in SGZ cell proliferation (39%, *P* = 3 × 10^−6^) in Cend1^−/−^ mice as indicated by Ki67 labeling (Figures 7J–L), as well as by an increase in DCX^+^/Ki67^+^ cycling neuroblasts (36%, *P* = 5.3 × 10^−5^) and in DCX^+^/Ki67^−^newborn neurons (31%, *P* = 3.39 × 10^−5^; Figures 7M–O). Concomitantly, there was a significant increase in cell apoptosis (60%, *P* = 0.006; Figures 7P–R).

Overall our analysis on adult hippocampal neurogenesis demonstrated that the earlier stages of this complex process, which include neural stem and progenitor cell activation as well as neuroblast and newborn neuron generation, are enhanced in the absence of Cend1. However at later stages, an increased number of cells are eliminated by apoptotic death, probably due to insufficient support provided by the reduced numbers of PV interneurons.

**Figure 8:**
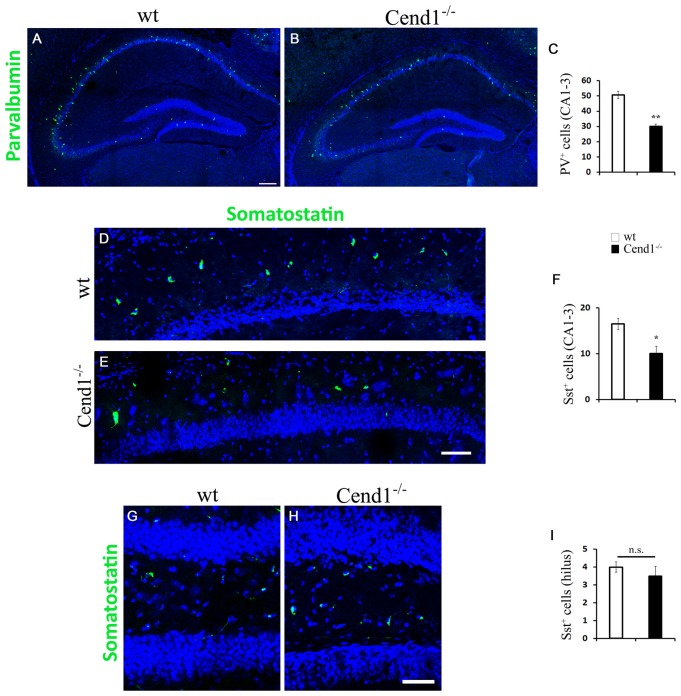
Decreased PV- and Sst-expressing interneurons in the hippocampal CA1–3 regions of adult Cend1^−/−^ mice. **(A,B)** Cryostat sections of the hippocampus, including the CA1–3 areas, immunostained for PV (green). Quantification **(C)** reveals a significant decrease of PV^+^ cells in the CA1–3 regions of Cend1^−/−^ mice (50.73 ± 2.42 PV^+^ cells in wt vs. 30 ± 1.52 in Cend1^−/−^ mice; *n* = 3 per genotype). **(D,E)** Immunofluorescence labeling for Sst (green) in CA1 and quantification in CA1–3 **(F)**, shows a significant decrease in this interneuron subtype in Cend1^−/−^ mice (16.5 ± 1.19 Sst^+^ cells in wt vs. 10 ± 1.58 in Cend1^−/−^ mice; *n* = 3 per genotype). **(G–I)** Sst^+^ cells in the hilus of the DG do not differ between wt and Cend1^−/−^ mice (4 ± 0.28 Sst^+^ cells in wt vs. 3 ± 0.5 in Cend1^−/−^ mice; *n* = 3 per genotype). Nuclei are seen with TOPRO3 (blue). Values represent means ± SEM. Scale bar **(A,E)**, 200 μm; **(H)**, 50 μm; *P < 0.05, **P < 0.01; n.s., non significant.

Finally, the reduction in PV interneurons noted in the DG prompted us to ask if there are also alterations in the abundance of these cells, as well as of Sst-expressing interneurons, all over the hippocampus. This is an important question given the involvement of these interneuron subtypes in memory processes (Murray et al., 2011; Ognjanovski et al., 2017). Accordingly, we found a reduction in both interneuron subpopulations in the CA1–3 regions of Cend1^−/−^ mice (41% reduction, *P* = 0.0054 for PV; 39%, *P* = 0.013 for Sst; Figure 8). However, unlike PV interneurons, we did not observe a similar reduction in the Sst^+^ population within the hilus of the DG in Cend1^−/−^ mice (Figure 8). This data in conjunction with the aberrant adult hippocampal neurogenesis underscore the contribution of Cend1 in learning and memory processes.

## Discussion

In this work we identified increased anxiety-related behavior and cognitive impairment in adult Cend1-deficient mice that were accompanied by cellular alterations in functionally relevant brain areas. These included a decrease in the density of GABAergic interneurons in the somatosensory cortex, the hippocampus and the amygdala, particularly in the basolateral nucleus, and aberrant hippocampal neurogenesis. The decrease in GABAergic interneurons in the cortex and amygdala identified in Cend1^−/−^ mice was associated with higher proliferation and apoptosis as well as reduced migration of neuronal progenitors from the embryonic GEs where these cells originate. Further, we showed that Cend1 specifically influences the generation and/or differentiation of GABAergic neurons since glutamatergic cells are not affected, at least in the cortex. These observations complement our previous studies suggesting that Cend1 is a molecular determinant of the neuronal lineage coordinating cell cycle exit and differentiation of neuronal precursors (Koutmani et al., 2004; Georgopoulou et al., 2006; Politis et al., 2007a,b). In support, gain- and loss-of-function experiments in neural stem/ progenitor cells have shown that Cend1 prompts neuronal precursors to exit the cell cycle and differentiate to a neuronal fate (Katsimpardi et al., 2008; Makri et al., 2010). Our first report on the characterization of Cend1^−/−^ mice revealed irregularities in cerebellar lamination that resulted in ataxic gait and deficits in motor coordination (Sergaki et al., 2010). Consistent with Cend1 function, these defects arose from enhanced proliferation of granule cell precursors, delayed radial migration of granule cells and compromised Purkinje cell differentiation. Collectively, our previous and current data highlight the necessity of Cend1 expression both during development and in the adult for establishment of a structurally and functionally normal phenotype.

GABA and glutamate are the two main neurotransmitters in the adult brain, which sustain the inhibitory-excitatory balance that is essential for proper function (Koós and Tepper, 1999; Markram et al., 2004; Xu et al., 2011; Takesian and Hensch, 2013). Inhibitory networks of GABAergic interneurons in the amygdala have a central role in shaping anxiety responses in the normal brain and at pathological states (Nuss, 2015). A decline in the GABAergic population resulting in reduced inhibitory neurotransmission in the amygdala is consistent with increased levels of anxiety (LeDoux, 2003), with the basolateral and central nuclei especially being involved in this process (Tye et al., 2011). Several lines of evidence indicate that additional brain circuits comprising top-down mechanisms originating in the cortex also modulate anxiety responses. Thus, in the somatosensory cortex, a reduction in local circuit inhibition due to lower levels of GABAergic interneurons was accompanied by increased anxiety (Matsuda et al., 2011). These findings suggest a causal relation between the increased anxiety-related behavior seen in Cend1^−/−^ mice and the decrease in GABAergic interneurons observed in both the basolateral nucleus of the amygdala and the somatosensory cortex. Interestingly, not all interneuron subtypes were equally affected in a given brain region. In the cortex of Cend1^−/−^ mice both PV and calbindin expressing neurons were reduced as well as Sst-expressing cells, which comprise a much smaller interneuron population. On the other hand in the BLA, only calbindin, but not PV, neurons were different between genotypes. Interestingly, while PV^+^ BLA neurons have been shown not to be implicated in emotional memory (Burghardt et al., 2006; Bienvenu et al., 2012; Butler et al., 2017), calbindin^+^ BLA neurons have been involved in processes underlying synaptic plasticity-mediated encoding of fear memory (Bienvenu et al., 2012).

Cend1^−/−^ mice, although exhibiting increased innate fear/anxiety, show reduced freezing following cued fear conditioning. This counter-intuitive behavior could be attributed to a malfunctioning feed-in circuit from sensory cortices to the amygdala. During cued fear conditioning, both the auditory and somatosensory cortices are activated and their proper function is GABA-tuned (Scaife et al., 2007). In order for the aversive association to be formed between the tone (conditioning stimulus-cue) and the noxious stimulus (unconditioned stimulus-pain caused by the electroshock), the two cortices (auditory and somatosensory) have to function efficiently and send convergent and precisely timed information to the BLA. If the timing and/or strength of incoming information are insufficient, the association fails to be formed and this is manifested as reduced freezing in future exposures to the conditioning stimulus. It should be noted that a similar behavioral profile with increased innate fear/anxiety and reduced learned (conditioned) fear has been observed in a transgenic mouse overproducing corticotropin-releasing hormone (van Gaalen et al., 2002).

Cend1^−/−^ mice display spatial learning and memory defects as determined in the MWM test. To exclude the possibility that this deficit was not associated with cognition but rather with their ataxic phenotype, we checked and confirmed that Cend1^−/−^ animals swam normally. This is not surprising, as in other models of ataxic mice, ataxia did not affect the swimming speed of the animals, possibly due to the nature of this measurement: gait-impaired animals can, apparently, compensate in the water with other body movements, or extra hind-limb kicks (Lalonde and Botez, 1986; Savvaki et al., 2008; Jayabal et al., 2015). The cellular changes in the somatosensory cortex and the hippocampus of Cend1^−/−^ mice should also contribute to the spatial learning and memory defects noted in these animals; while it is the hippocampus that subserves acquisition and recall of new spatial information, memory storage is eventually mediated by the cortex, with modulation of gene expression in various areas, including the somatosensory, but not the motor, cortex (Park et al., 2011). Particularly regarding the role of PV-positive GABAergic interneurons, it has been shown that a selective inactivation of this sub-population in the cortex affects negatively only cognitive abilities, suggesting that these interneurons may contribute to specific functions of cortical circuits (Murray et al., 2015). Such a divided role between different interneuron populations is further supported by studies showing that targeting of the Sst-positive population of interneurons in the prefrontal cortex of Sst-Cre mice, had no effect on working memory in a task afflicted by the inactivation of PV-expressing interneurons (Taniguchi et al., 2011).

Cend1^−/−^ mice also display aberrations in hippocampal neurogenesis, which is particularly interesting because of its association with higher cognitive functions, particularly memory processes. In agreement with Cend1 function in other brain areas, we noted a cell proliferation increase in the DG SGZ in Cend1^−/−^ mice, resulting in higher numbers of neural stem and progenitor cells as well as in increased numbers of cycling neuroblasts and newborn neurons. These features were paralleled by an increase in local astrocytes, which are known to stimulate neurogenesis (Song et al., 2002), and a decrease in local PV-expressing interneurons. It has been previously reported that PV-expressing interneurons provide immature GABAergic synaptic inputs to proliferating adult mouse hippocampal neural precursors from. Through this mechanism, PV cells suppress the activation of quiescent neural stem cells (Song et al., 2012) while at later stages of neurogenesis they promote survival of neuronal progeny (Song et al., 2013). Therefore, the enhancement of the earlier stages of neurogenesis in Cend1^−/−^ mice may be attributed to cell intrinsic mechanisms and/or to cell extrinsic influences arising from the dual effects of an increase in local astrocytes and a decrease in PV interneurons. At the same time, increased cell elimination by apoptotic death may be due to insufficient support provided by the reduced numbers of PV interneurons.

These combined effects might affect the maintenance of a normal neuronal circuitry in the adult hippocampus, contributing to the cognitive impairment of Cend1^−/−^ mice. Apart from their regulatory role in neurogenesis, PV^+^ GABAergic neurons innervate the cell body of CA1 pyramidal neurons and control their glutamatergic output (Klausberger and Somogyi, 2008). It has been shown that selective removal of PV-expressing neurons leads to impaired spatial memory (Murray et al., 2011), and therefore the decrease in PV^+^ neurons may disturb the overall output, leading to memory defects in Cend1^−/−^ mice.

The population of hippocampal Sst neurons shows an area-dependent effect in Cend1−/− mice, being decreased in CA1–3 areas while not being affected in the DG. This difference could be attributed to differences in their developmental profile, as it has been shown that Sst neurons in CA1–3 are born before those in the DG (Rapp and Amaral, 1988). The differential effect of Cend1 ablation on PV and Sst DG neurons is interesting given the distinct role of these inhibitory interneurons in the flow of input from cortical areas to other hippocampal areas, with PV neurons regulating the spiking of DG granule cells while both populations are involved in containing granule cell activity (Lee et al., 2016). Nevertheless, a precise tuning of the functions of both types of interneurons is necessary for proper DG function e.g., during memory formation for filtering out irrelevant information. In contrast to Sst interneurons, the PV neuronal population was decreased throughout the hippocampus, a phenomenon that could also be related to developmental differences of these two types of interneurons; for instance, Sst neurons are produced during the first half of neurogenesis, while PV neurons show a protracted period of birth throughout the neurogenic period (Inan et al., 2012).

In conclusion, the behavioral and cellular alterations we show in the present study elucidate previously unknown aspects of Cend1 involvement during development and in the adult and together with previous data underline the importance of Cend1 expression for the establishment of a structurally and functionally normal phenotype.

## Author Contributions

AS, AL, FS and RM conceived the study. KS, AS and AL designed and performed the experiments; analyzed and interpreted data with the contribution of FS and RM. KS, AS and RM wrote the article with the contribution of FS and AL. RM and FS provided financial support. All authors approved the final manuscript.

## Conflict of Interest Statement

The authors declare that the research was conducted in the absence of any commercial or financial relationships that could be construed as a potential conflict of interest.

## Supplementary Material

FIGURE S1 No difference in the density of glutamatergic projection neurons in the adult cortex of Cend1^−/−^ mice. Confocal images of cryostat sections of adult somatosensory cortex immunostained for Satb2 **(A)** and quantification of Satb2^+^ cells in Cend1^−/−^ mice as compared to wt animals **(B)**. Values represent mean ± SEM. Scale bar **(A)**, 100 μm.

FIGURE S2 No evidence for inflammatory microgliosis. Double labeling for the microglial/macrophage marker Iba1 (red) and the activated microglial/macrophage marker CD68 (green) shows no signs of inflammatory microgliosis in the hippocampal DG of Cend1^−/−^ mice as compared with wt mice. Nuclei are counterstained with TOPRO3. Scale bar 100 μm.

## Abbreviations

BLA: basolateral amygdala
CB: calbindin
DCX: doublecortin
DG: dentate gyrus
EPM: elevated plus maze
GABA: γ- amino butyric acid
GE: ganglionic eminence
GFAP: glial fibrillary acidic protein
MGE: medial ganglionic eminence
MWM: Morris water maze
NDS: normal donkey serum
PBS: phosphate-buffered saline
PH3: phosphohistone 3
PV: parvalbumin
SGZ: subgranular zone
Sst: somatostatin
wt: wild type.
