# Supplementary figures and images for: Increased Anxiety-Related Behavior, Impaired Cognitive Function and Cellular Alterations in the Brain of Cend1-deficient Mice
Source: Front Cell Neurosci. 2019 Jan 29;12:497. doi: 10.3389/fncel.2018.00497 (PMC6361865; doi:10.3389/fncel.2018.00497)

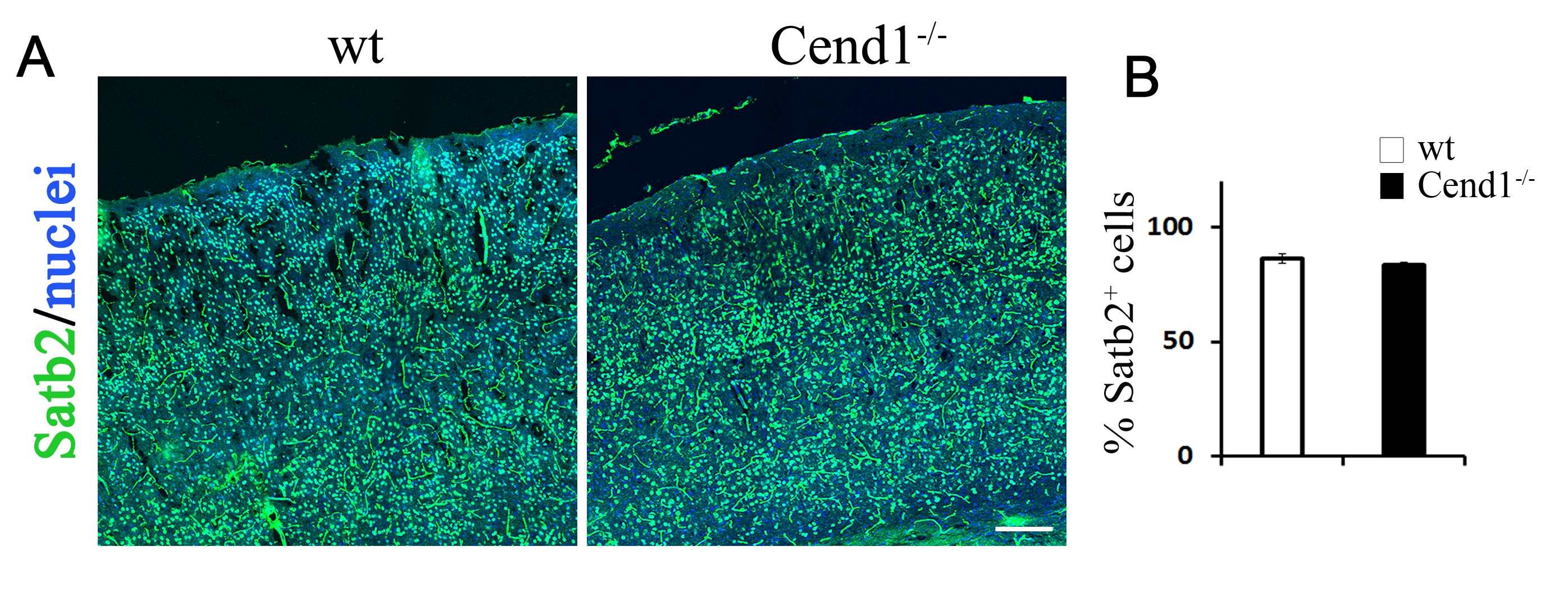

Supplement: FIGURE S1 — No difference in the density of glutamatergic projection neurons in the adult cortex of Cend1−/− mice. Confocal images of cryostat sections of adult somatosensory cortex immunostained for Satb2 (A) and quantification of Satb2+ cells in Cend1−/− mice as compared to wt animals (B). Values represent mean ± SEM. Scale bar (A), 100 μm. [file Image_1.TIF]

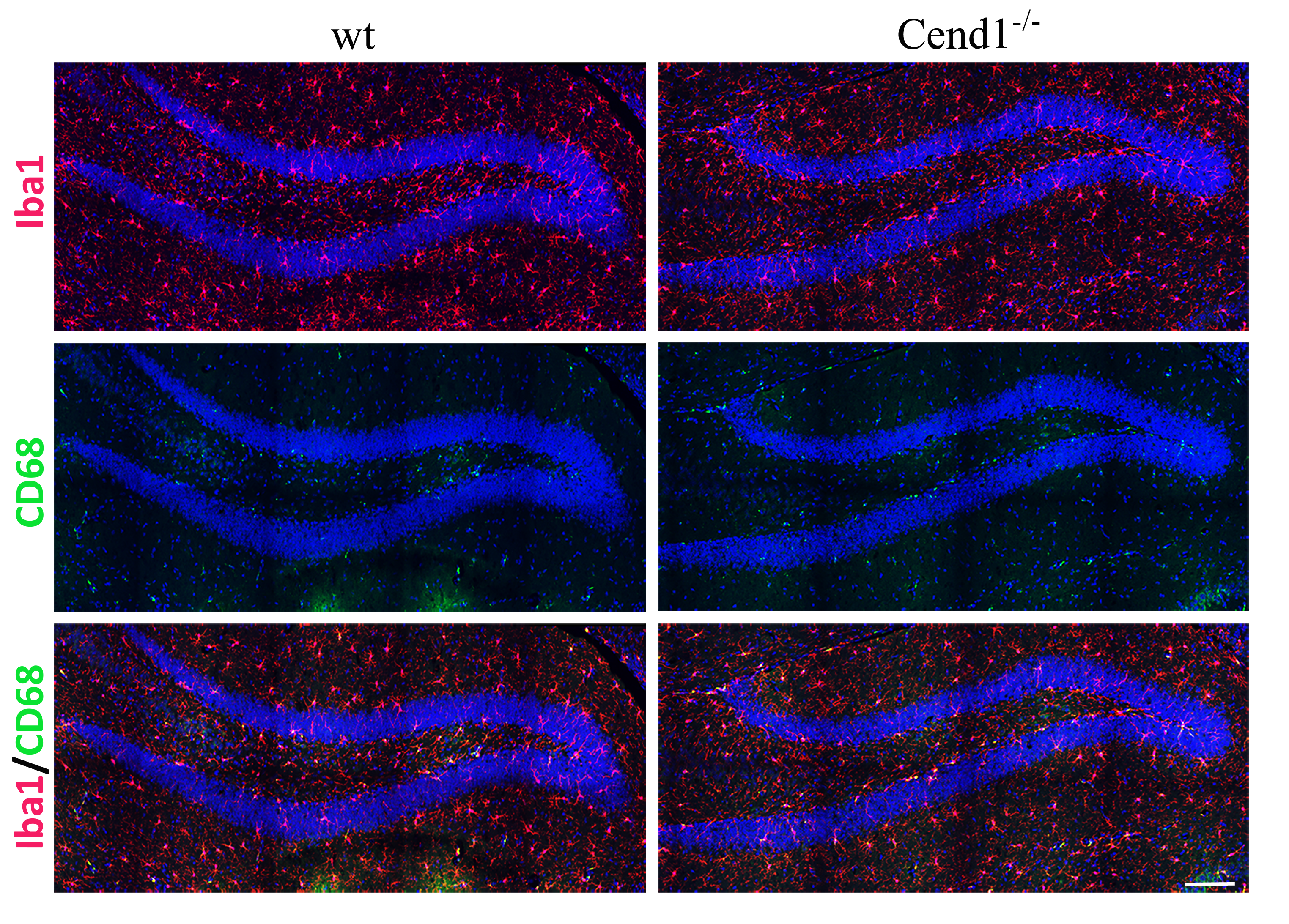

Supplement: FIGURE S2 — No evidence for inflammatory microgliosis. Double labeling for the microglial/macrophage marker Iba1 (red) and the activated microglial/macrophage marker CD68 (green) shows no signs of inflammatory microgliosis in the hippocampal DG of Cend1−/− mice as compared with wt mice. Nuclei are counterstained with TOPRO3. Scale bar 100 μm. [file Image_2.TIF]
